# Capillary Leak Syndrome With Pulmonary Edema Preceded by Organizing Pneumonia Caused by Combination Therapy With Nivolumab and Ipilimumab: A Case Report

**DOI:** 10.1016/j.jtocrr.2023.100491

**Published:** 2023-02-24

**Authors:** Hiroaki Tachi, Atsuhito Shibagaki, Shu Teshima, Midori Hanazawa, Shihori Matsukura, Kei Shimizu, Yusuke Yamamoto

**Affiliations:** Department of Respiratory Medicine, Hitachi General Hospital, Hitachi City, Japan

**Keywords:** Immune checkpoint inhibitor, Drug-induced lung injury, Organizing pneumonia, Capillary leak syndrome, Pulmonary edema, Case report

## Abstract

Treatment with drugs can cause lung disorders. Immune checkpoint inhibitors are often associated with organizing pneumonia. Capillary leak syndrome is a clinical form of drug-induced lung injury, a rare condition characterized by hemoconcentration, hypoalbuminemia, and hypovolemic shock. There have been no reports of multiple lung injury with immune checkpoint inhibitors, and although capillary leak syndrome alone has been reported in the past, there have been no reports of pulmonary edema as a complication. We report a 68-year-old woman who died of respiratory and circulatory failure owing to pulmonary edema caused by capillary leak syndrome, preceded by organizing pneumonia induced by combination therapy with nivolumab and ipilimumab for postoperative recurrence of lung adenocarcinoma. Residual inflammation and immune abnormalities from previous immune-related pulmonary adverse events may have increased pulmonary capillary permeability, leading to marked pulmonary edema.

## Introduction

When drug-induced lung injury occurs, it is useful to first identify the imaging pattern to predict prognosis, as treatment response varies with clinical forms. It is common for one drug to cause one pulmonary adverse event, and it is rare for a single therapeutic intervention to cause multiple imaging patterns over time. Furthermore, although a pattern of pulmonary edema owing to capillary leak syndrome (CLS) is known as a clinical form of drug-induced lung injury,^1^ there are no reports of that caused by immune checkpoint inhibitors (ICIs).

## Case Presentation

**Figure 1:**
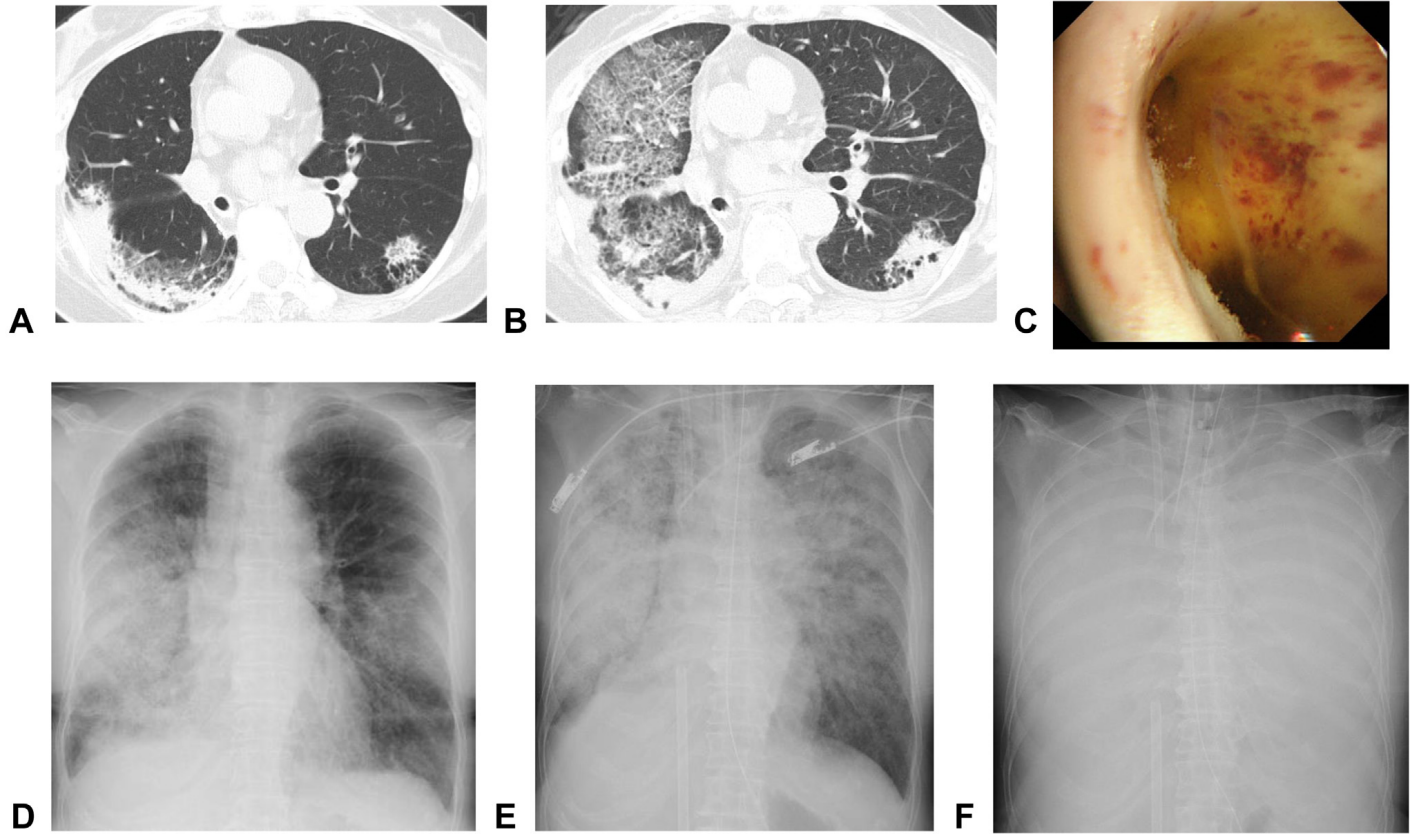
(*A*) Chest CT on day 43 revealed infiltrative shadows with contraction tendency in both lung lobes. (*B*) Chest CT on day 87 revealed extensive consolidation and ground-glass opacity in both lung lobes. (*C*) The bronchial lumen at the junction of the right upper lobe branch and the middle bronchial trunk was filled with yellow fluid. (*D*) Chest radiograph on day 87 (time of admission) revealed consolidation in both lung fields. (*E)* Chest radiograph on day 87 (8 hours after admission) revealed rapid deterioration of consolidation in both lung fields. (*F*) Chest radiograph on day 87 (16 hours after admission) revealed a complete loss of permeability in both lung fields. CT, computed tomography.

We present a 68-year-old woman with no substantial medical history. A chest radiograph at physical examination revealed a nodule shadow in the right lower lung field. Bronchoscopy revealed mucinous adenocarcinoma. After right lower lobectomy and lymph node dissection, the patient was diagnosed as pT4N0M0 stage IIIA with no driver gene mutation and no expression of programmed death-ligand 1. After three courses of cisplatin and vinorelbine as postoperative adjuvant chemotherapy, she was referred to our department for chemotherapy for postoperative recurrence owing to multiple lung metastases on chest computed tomography (CT). Nivolumab (anti–programmed cell death protein-1) and ipilimumab (anti–CTLA-4) combination therapy were started. On the forty-third day of treatment, chest CT revealed infiltrative shadows with contraction tendency in both lung lobes, and bronchoalveolar lavage revealed increased total cell count with eosinophil predominance (Table 1). She was diagnosed as having an immune-related lung adverse event with a pattern of organizing pneumonia. As the patient was asymptomatic and the shadows were mild, drug administration was ceased. However, on day 48, the consolidations were enlarged (Fig. 1*A*), so prednisolone 60 mg was started, and the dose was then reduced to 60 mg (d 49–55), 40 mg (d 56–62), 30 mg (d 63–69), 20 mg (d 70–77), and 15 mg (d 78–84), as the lung damage was improving. On day 85, prednisolone was tapered to 10 mg; however, dyspnea on exertion appeared on the same day. On day 87, the patient visited our outpatient clinic and required hospitalization with ventilator management because of acute respiratory failure. Chest CT revealed extensive consolidation and ground-glass opacity in both lung lobes (Fig. 1*B*), and bronchoscopy revealed a bronchial lumen filled with yellow fluid (Fig. 1*C*). Cytology and microbiologic results were negative, and she had an increased total cell count with the predominance of neutrophils (Table 1). Echocardiography revealed no abnormalities in cardiac function, but the patient presented with hypovolemic shock, and despite a massive infusion of fluids, no improvement in circulatory status was achieved. We administered antibiotics, cyclophosphamide, and steroid treatment with 1000 mg methylprednisolone per day, but the patient died of worsening respiratory failure owing to rapidly decreased permeability of both lung fields (Fig. 1*D*–*F*). In retrospect, she was considered to have drug-induced CLS because hemoconcentration and hypoalbuminemia were observed on blood tests (Table 2).

**Table 1:** Findings of Bronchoalveolar Lavage Fluid

	Initial Examination	Second Examination
Bronchus	rt.B^5^b	rt.B^3^b
Recover rate, %	30.0	47.3
Total cell count	3.06 × 10^5^/mL	3.30 × 10^5^/mL
Macrophages, %	79	26
Neutrophils, %	2	65
Lymphocytes, %	11	6
Eosinophils, %	8	3
Basophils, %	0	0
CD4-to-CD8 ratio	0.60	0.21
Culture	Negative	Negative
Cytology	ClassⅠ	ClassⅠ

**Table 2:** Blood Pressure and Blood Test Results Before and After Hospitalization

	Prior to Admission	At the Time of Admission	The Day After Admision
Blood pressure （mm Hg）	112/61	82/68	114/86
RBC（×10^5^/μL）	3.87	555	399
Hb（g/dL）	10.8	15.6	11.1
Ht（%）	33.7	47.8	33.9
ALB（g/dL）	3.5	3.4	2.2
BUN（mg/dL）	19.7	20.1	22.2
Cre（mg/dL）	0.74	0.87	1.05

ALB, albumin; BUN, blood urea nitrogen; Cre, creatinine; Hb, hemoglobin; Ht, hemorrhagic transformation; RBC, red blood cell.

Underline indicates data showing hypotension and hemoconcentration.

## Discussion

To the best of our knowledge, this is the first case report of noncardiogenic pulmonary edema and hypovolemic shock owing to CLS, preceded by organizing pneumonia caused by combination therapy with nivolumab and ipilimumab. This case report is very valuable for two reasons: the immune-related adverse events (irAEs) presented multiple imaging patterns in one individual, and the CLS caused by ICIs resulted in pulmonary edema, which has not been reported previously.

In lung cancer immunotherapy, programmed cell death protein-1 inhibitors, such as nivolumab, and CTLA-4 inhibitors, such as ipilimumab, play an important role in tumor control by activating CD8 cells and disabling regulatory T cells. However, they can cause the development of excessive autoimmunity in various organs. Although a variety of imaging findings have been reported for drug-induced lung injury owing to ICIs, the most common pattern is consolidation and ground-glass opacity suggestive of organizing pneumonia.^2^ CLS is known as a pulmonary edema pattern, but it is an uncommon finding.^1^ Several cases of CLS caused by ICIs for lung cancer have been reported^3^; however, none resulted in noncardiogenic pulmonary edema. There is no knowledge of whether the combination therapy with nivolumab and ipilimumab increases the risk of CLS more than nivolumab alone, but a higher incidence of pulmonary irAEs induced by the combination therapy has been reported.^4^ Although the exact pathogenesis of CLS is still unclear, it is thought to be caused by increased vascular permeability owing to dysfunction of the vascular endothelium, resulting in leakage of plasma and proteins into the interstitium.^5^ In the present case, the patient first developed lung damage in the pattern of organizing pneumonia as an irAE. The second bronchoalveolar lavage revealed a change from eosinophil predominance to neutrophil predominance, suggesting a different pathogenesis from the initial irAE, and the clinical course led to the diagnosis of CLS. Although there has been no established evidence of steroid reduction methods for drug-induced lung injury, the rate of reduction may have been relatively fast in this case (from 60 mg to 10 mg in 5 wk). In addition, it has been reported that CD8 cells surrounded the damaged endothelial cells of systemic CLS.^6^ Thus, it is possible that the residual inflammation caused by the relatively rapid tapering of prednisolone for drug-induced organizing pneumonia, combined with immune abnormalities involving CD8 cells, increased vascular permeability in the lungs, leading to CLS.

## Conclusion

Drug-induced CLS should be considered as a differential disease when unexplained pulmonary edema occurs during treatment with ICIs, even more so in cases of a previous irAE pulmonary injury.

## CRediT Authorship Contribution Statement

**Hiroaki Tachi:** Writing—original draft.

**Atsuhito Shibagaki, Shu Teshima, Midori Hanazawa, Shihori Matsukura:** Data curation, Visualization.

**Kei Shimizu:** Conceptualization.

**Yusuke Yamamoto:** Supervision, Writing—review and editing.
